# Requirement of *lmo1930*, a Gene in the Menaquinone Biosynthesis Operon, for Esculin Hydrolysis and Lithium Chloride Tolerance in *Listeria monocytogenes*

**DOI:** 10.3390/microorganisms7110539

**Published:** 2019-11-08

**Authors:** Cameron Parsons, Midya Jahanafroozi, Sophia Kathariou

**Affiliations:** Department of Food, Bioprocessing and Nutrition Sciences, North Carolina State University, Raleigh, NC 27606, USA

**Keywords:** esculin, *Listeria monocytogenes*, menaquinone, selective enrichment

## Abstract

*Listeria monocytogenes* is a foodborne pathogen that is widely distributed in nature, having been isolated from a variety of sources such as soil, water, plant matter, and animals. In addition, *L. monocytogenes* is often detected in the regular sampling of food and food processing environments. The most common method for detecting *L. monocytogenes* is the use of selective enrichments. Both lithium chloride and esculin, in combination with ferric ammonium citrate, are utilized in several of the most commonly-employed selective enrichment schemes for *L. monocytogenes*. Here we report that transposon-based inactivation of *lmo1930,* one of the genes in the menaquinone biosynthesis operon, via transposon mutagenesis severely impaired the ability of *L. monocytogenes* to grow in the presence of lithium chloride or hydrolyze esculin, and conferred reduced growth and colony size. All phenotypes were restored upon genetic complementation. Thus, strains of *L. monocytogenes* with mutations leading to inactivation of *lmo1930* may evade many commonly-used selective enrichment protocols employed in the detection of *L. monocytogenes*.

## 1. Introduction

*Listeria monocytogenes* is found ubiquitously in nature and is a facultative intracellular pathogen, capable of causing severe disease and death in susceptible individuals [1,2]. For these reasons, the rapid and reliable detection of *L. monocytogenes* is a key factor in the surveillance of this pathogen. Selective enrichment is typically used to detect and isolate *L. monocytogenes* from food and environmental samples. While there are numerous validated selective enrichment protocols for *L. monocytogenes* [3,4,5], many of these approaches share ingredients such as lithium chloride and esculin in combination with ferric ammonium citrate to differentiate *L. monocytogenes* from other microorganisms.

Esculin is a biopolymer that is widely distributed in several types of plants [6]. Given its presence in soil and plant matter, the ability to utilize this biopolymer may be useful for environmental survival of *L. monocytogenes*. The ability to hydrolyze esculin has been observed in diverse microorganisms and esculin has also been incorporated as a differential-selective agent into several selective enrichment protocols for *L. monocytogenes*. Esculin hydrolysis in the presence of ferric ammonium citrate results in a black precipitate that is easily detected with the naked eye in either liquid or solid media, allowing detection of putative *L. monocytogenes* or other *Listeria* spp. on agar media such as Modified Oxford (MOX) [7,8]. If esculin hydrolysis or lithium chloride tolerance were compromised, *L. monocytogenes* and other *Listeria* spp. would be undetectable in many of the currently employed enrichment protocols, with potentially important food safety and public health implications. In this work we investigated the involvement of *lmo1930* in esculin hydrolysis and lithium chloride tolerance in *L. monocytogenes* and assessed the importance of this gene for identification of *L. monocytogenes* in selective enrichment protocols that utilize esculin and lithium chloride. We employed transposon mutagenesis and genetic complementation to identify and characterize a gene essential in esculin hydrolysis in *L. monocytogenes* strain 2010L-1723, implicated in an outbreak of listeriosis via contaminated celery.

## 2. Methods

### 2.1. Bacterial Strains and Growth Conditions

Unless otherwise specified, strains were routinely cultured in Trypticase soy broth (Becton, Dickinson and Co (BD), Sparks, MD, USA) supplemented with 0.7% yeast extract (BD) (TSBYE) or in brain heart infusion (BHI; BD). TSBYE or BHI with 1.2% agarose (BD) was used for solid media (TSAYE and BHIA, respectively). When appropriate, media were supplemented with esculin (1 g/L) (Acros Organics, Fair Lawn, NJ, USA) and ferric ammonium citrate (0.5 g/L) (MP Biomedicals, Solon, OH, USA). Growth was assessed aerobically on TSAYE and BHIA with and without esculin/ammonium ferric citrate, as well as modified oxford medium (BD) (MOX) and RAPID’L.mono (Biorad, Marnes-la-Coquette, France). In addition, growth was assessed anaerobically at 37 °C on MOX, TSAYE, and BHIA using an anaerobic chamber (Coy Laboratory Products Inc., Grass Lake, MI, USA). To assess tolerance to lithium chloride (LiCl), dilutions of overnight cultures grown in BHI were plated on BHIA supplemented with 15 g/L LiCl (Amresco, Solon, OH, USA) and monitored for growth at 37 °C for 48 h. To assess the role of menaquinone in relation to esculin hydrolysis, strains were streaked on TSAYE supplemented with esculin, ferric ammonium citrate, and 50 mg of vitamin K_2_/liter (Supelco, Bellefonte, PA, USA). The plates were then incubated over 48 h at 37 °C and observed for growth and the formation of black precipitate. To assess relative growth levels in BHI and TSBYE, growth was monitored over 48 h at 37 °C in a Bioscreen C absorbance microplate reader (Oy Growth Curves Ab Ltd., Raisio, Finland). Growth curves were constructed from four biological replicates in one technical trial for each strain. Recorded changes in optical density (600 nm) were used to plot a growth curve for each strain in BHIB and TSBYE.

### 2.2. Mutant Library Construction and Mutant Screening

Mariner-based transposon mutant libraries, consisting of ~3000 individual mutants, were constructed as previously described [9,10], in *L. monocytogenes* strains 2011L-2858 from the 2011 cantaloupe outbreak [11], and 2010L-1723 from a 2010 outbreak associated with celery [12]. Individual mutants were screened on TSAYE supplemented with esculin and ammonium ferric citrate. The transposon insertion in a mutant lacking the ability to hydrolyze esculin was localized using arbitrary PCR as described previously [9,10]. PCR primers flanking the putative insertion site were lmo1930F (5′-CTGATAATCGGCTTCACTTTG-3′) and lmo1930R (5′-GCAGCTGCTGACACT ACTT-3′). The subsequent PCR product was then visualized on a gel and sequenced (Genewiz Inc., South Plainfield, NJ, USA). The copy number of the transposon was established using qPCR as described [13].

### 2.3. Genetic Complementation

**Figure 1:**
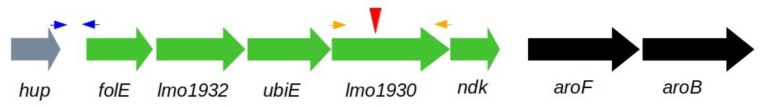
Organization of the *lmo1930* genomic region in *L. monocytogenes* EGD-e. Genes belonging to the *lmo1930* operon as described by Toledo-Arana et al. (2009) are indicated in green. Downstream genes *aroF* and *aroB* belonging to the *aroF aroB lmo1926 hisC tyrA aroE* operon (Toledo-Arana et al. (2009) are in black. The transposon insertion in *lmo1930* is indicated with a downwards-pointing red triangle. Primers H1H3compsoeA and H1H3compsoeB used for amplification of the upstream regulatory region are indicated with blue arrows. Primers H1H3compsoeC and H1H3compsoeD used for amplification of the *lmo1930* coding region are indicated with orange arrows.

The coding region of the *lmo1930* homolog in strain 2010-1723 (hereafter referred to as “*lmo1930*”) was amplified using primers H1H3compsoeC (5′-TAAAAAGATAGGGGAATTAAGGATGAAACTTAACT TTTTATATGC-3′) and H1H3compsoeD (5′-CAGTGCGGCCGCTTAATAATTTCTTTTATCTAAAAC AC-3′). Since *lmo1930* is located within an operon (Figure 1), the upstream region at the start of the operon that contained the *in silico*-predicted promoter was also amplified using primers H1H3compsoeA (5′-GTACGGTACCGCTACATAGACTTTTAAGCACT-3′) and H1H3compsoeB (5′-CCTTAATTCCCCTATCTTTTTA-3′). These two PCR products were then purified and joined together using splicing by overlap extension (SOE) PCR as previously described [14], to provide a fusion of the upstream regulatory region with the coding region of *lmo1930* from strain 2010L-1723. The resulting product was digested with KpnI and NotI (New England Biolabs, Ipswitch, Ma, USA) and ligated into similarly-digested shuttle vector pPL2 [15]. The resulting recombinant plasmid pPLH1H3comp was transformed into chemically-competent *Escherichia coli* DH5α (Invitrogen, Waltham, MA, USA), resulting in strain *E. coli* DH5*α*_pPLH1H3comp. It was then extracted from *E. coli* DH5*α*_pPLH1H3comp and electroporated into mutant H1H3, resulting in strain H1H3::lmo1930.

## 3. Results and Discussion

### 3.1. Insertional Inactivation of lmo1930 Abolished Esculin Hydrolysis and Resulted in Impaired Growth

**Figure 2:**
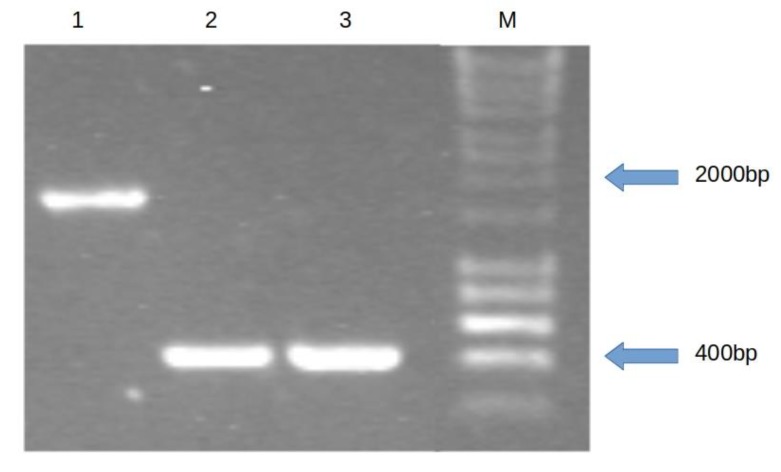
PCR confirmation of the transposon insertion in *lmo1930.* Bands are the result of amplification with lmo1930F and lmo1930R as described in Materials and Methods. Lanes: 1, mutant H1H3; 2, parental strain 2010L-1723, 3, genetically-unrelated, serotype 1/2b strain 2011L-2858; M, molecular weight markers (HyperLadder 1kb; Bioline, Boston, MA, USA).

The capacity to hydrolyze esculin was determined for 2392 and 1092 mariner-based transposon mutants of *L. monocytogenes* strains 2011L-2858 and 2010L-1723, respectively. The mutants were tested on TSAYE supplemented with esculin and ferric ammonium citrate (TSAYE-Es) to identify those unable to hydrolyze esculin and thus, lacking black pigmentation on the agar. This screening identified one esculin hydrolysis-deficient mutant of strain 2010L-1723, designated H1H3, while similar mutants of 2011L-2858 were not identified. Arbitrary PCR indicated that H1H3 harbored a transposon insertion in *lmo1930* (Figure 1). PCR with primers flanking this site resulted in a product that was ~1.4 kb (the approximate size of the mariner-based transposon) larger than the PCR product from the parental strain (Figure 2). Sequencing of this PCR product localized the transposon insertion at nt 595 in the coding region of the *lmo1930* homolog of 2010L-1723. Real-time PCR indicated that this mutant harbored a single copy of the transposon (data not shown).

**Figure 3:**
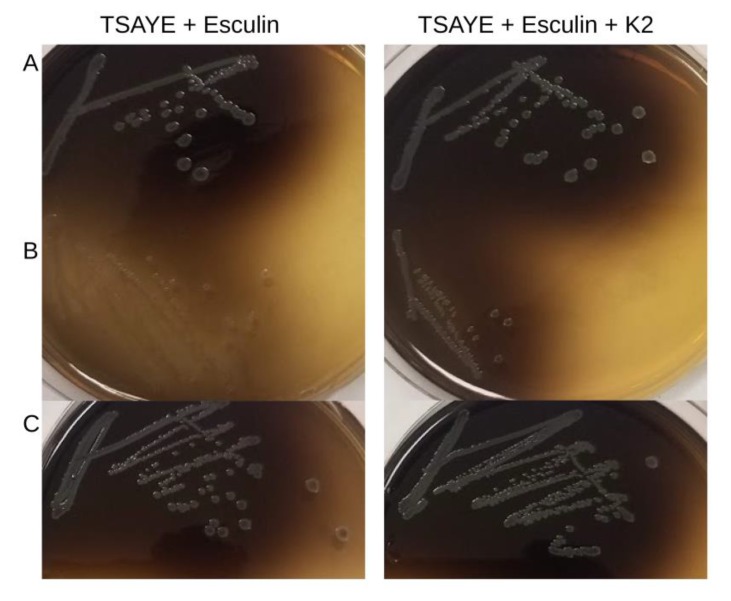
Impact of the transposon insertion in *lmo1930* on colony size and esculin hydrolysis of *L. monocytogenes*, and effects of K2 supplementation. Cultures were grown on media with esculin / ferric ammonium citrate (TSAYE-Es) (left) or TSAYE-ES with vitamin K2 (right) as described in Materials and Methods. Panels: A, parental strain 2010L-1723; B, mariner-based mutant H1H3; C, H1H3 complemented with the *lmo1930* homolog. Strains were grown at 37 °C for 24 h (top) and 48 h (bottom).

**Figure 4:**
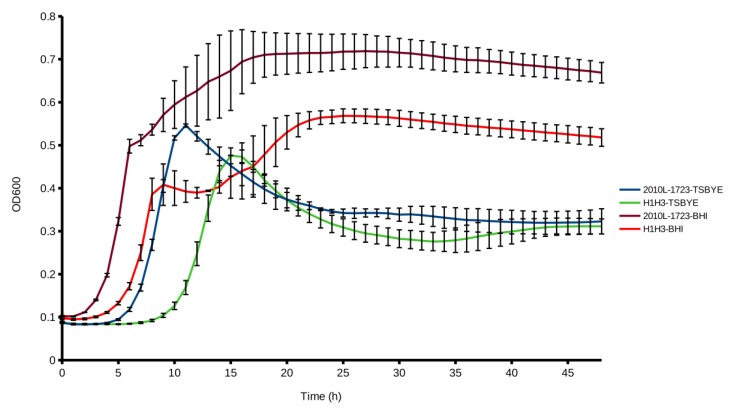
Aerobic growth of 2010L-1723 and H1H3 at 37 °C. Parental strain 2010L-1723 was grown in TSBYE (blue line) or BHI (orange line), and H1H3 was also grown in TSBYE (purple line) or BHI (green line) as described in the Material and Methods. Curves were generated from repeated measures of OD_600_ over 48 h. Each line represents the average measures of four biological replicates per strain in one representative trial. Error bars represent standard deviation.

On TSAYE-Es, H1H3 was unable to hydrolyze esculin, producing white colonies that were also consistently smaller than the parental strain (Figure 3). Smaller colonies were also produced on TSAYE (data not shown). Furthermore, H1H3 grew slower, with a prolonged lag phase in the non-selective liquid media TSBYE (Figure 4), and markedly lower final absorbance in BHI (Figure 4). Surprisingly, however, on BHI agar supplemented with esculin and ferric ammonium citrate, H1H3 was able to hydrolyze esculin and formed black colonies, even though the colony size was still considerably smaller than the parental strain (data not shown). Interestingly, this same behavior was observed in TSAYE supplemented with vitamin K2 (Figure 3). This finding suggests that nutrient components in BHI and vitamin K2 complement the esculin hydrolysis phenotype of the mutant that was noted in TSAYE-based media. The fact that H1H3 was capable of esculin hydrolysis on TSAYE supplemented with vitamin K2 suggests that menaquinone production was impacted in H1H3 and is likely linked to the esculin hydrolysis phenotype. However, BHI and vitamin K2 supplementation under the conditions that were employed appear to be inadequate for full restoration of colony size, possibly indicating that *lmo1930* is required for the production of additional compounds that are needed for wild type levels of growth. The findings also suggest that the mutant would likely not have been identified had the transposon library been screened on BHIA with esculin and ferric ammonium citrate, instead of TSAYE-Es.

### 3.2. Genetic Complementation Confirms Role of lmo1930 in Esculin Hydrolysis

Genetic complementation of *lmo1930* fused with its upstream regulatory region restored both colony size and esculin hydrolysis (Figure 3). This indicates that both of the observed phenotypes, i.e., reduced colony size and lack of esculin hydrolysis were both due to the inactivation of *lmo1930*. Furthermore, the complementation data demonstrate that these phenotypes were not due to potential polar effects of the transposon insertion on the previously-studied *aroB* or other genes downstream of *lmo1930.*

### 3.3. Inactivation of lmo1930 Markedly Reduces Tolerance to LiCl and Compromises Growth of L. Monocytogenes on LiCl-Containing Selective Media Such as MOX and RAPID’L.mono agar

**Figure 5:**
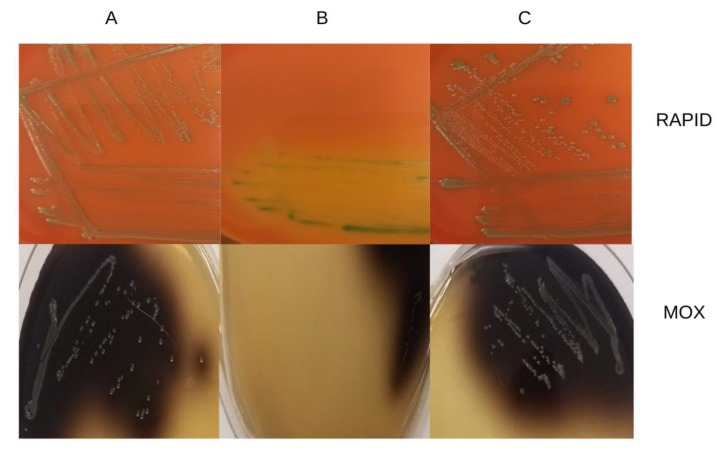
Growth of *L. monocytogenes* strains on RAPID’L.mono and MOX plates. A, parental strain 2010L-1723; B, mariner-based mutant H1H3; C, H1H3 complemented with the *lmo1930* homolog. Cultures were grown at 37 °C for 48 h as described in Materials and Methods.

As discussed above, esculin hydrolysis plays a key role in the differential detection of *L. monocytogenes* and other *Listeria* spp. in a number of selective media. MOX is one such medium that takes advantage of the black precipitate formed when esculin is hydrolyzed. Interestingly, we found that H1H3 not only failed to form back colonies but also lacked the ability to grow on MOX. The ability of H1H3 to grow on MOX was restored upon complementation with the intact *lmo1930* (Figure 5).

To identify which component(s) of MOX resulted in this lack of growth, H1H3 was tested for growth on BHIA with and without the MOX antimicrobial supplement, as well as on MOX base lacking the antimicrobial supplement. Growth of H1H3 was similar on BHIA with or without the MOX supplement (data not shown), suggesting that the MOX supplement was not a major contributor to the lack of growth of H1H3 on MOX.

MOX base contains LiCl (final concentration, 1.5%) as an agent to inhibit background microorganisms. Lithium is a chaotropic agent that has been shown to interfere with protein solubility [16], and we, therefore, hypothesized that the growth impairment of H1H3 on MOX may reflect impaired tolerance of the mutant to lithium. Indeed, when H1H3 was plated on BHI supplemented with 1.5% LiCl (BHI-LiCl), no growth was observed after 24 h, while the parental strain grew, albeit with smaller colonies than on BHIA (data not shown). By 48 h there was a four-log reduction in the population of H1H3 on BHIA-LiCl vs BHIA. This finding reveals a novel role of *lmo1930* in LiCl tolerance of *L. monocytogenes;* impaired tolerance to LiCl was a major contributor to the lack of growth of the *lmo1930* transposon mutant on MOX.

Besides MOX, LiCl is a key selective agent in several other *Listeria* selective agar media including LPM, PALCAM, RAPID’L.mono agar, and Brilliance *Listeria* agar, as well as Fraser and Half-Fraser broths used as primary/secondary enrichments in the commonly-employed ISO method [8,17]. Growth of H1H3 on RAPID’L.mono plates was severely inhibited with barely-visible growth, where the culture was most heavily inoculated on the agar plate and no individual colonies could be detected (Figure 5). The collective findings suggest that validated *L. monocytogenes* isolation methods that utilize LiCl would likely fail to recover strains with mutations in *lmo1930*.

**Figure 6:**
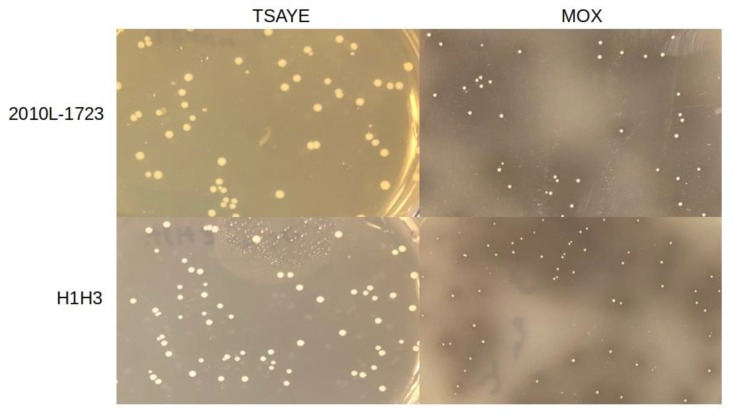
Anaerobic growth of 2010L-1723 and H1H3 under anaerobic conditions. Left panel; TSAYE; right panel, MOX. Strains were grown for 72 h at 37 °C as described in Materials and Methods.

Previously-characterized menaquinone biosynthesis mutants were shown to have primarily anaerobic metabolism [18]. In rich media such as BHI, inactivation of *aroA* led to reduced growth rates aerobically and anaerobically, while *aroB* mutants grew poorly aerobically and failed to grow at all anaerobically, and growth of an *aroE* mutant was severely impaired aerobically but improved anaerobically, albeit still impaired in comparison to the parental strain [18]. Colony size of H1H3 on BHIA or MOX was noticeably improved anaerobically, and the difference in colony size between H1H3 and the parental strain 2010L-1723 was not as striking anaerobically as under aerobic conditions, where in fact H1H3 was completely unable to grow on MOX (Figure 6). Further biochemical and transcriptional analysis is needed to assess whether metabolism in H1H3 is primarily anaerobic and especially impaired in the presence of oxygen.

The gene investigated here, *lmo1930* was not previously reported to be implicated in menaquinone biosynthesis. However, the *lmo1930*-inactivated mutant H1H3 exhibited phenotypes such as smaller colony size and growth deficiency in BHI. A similar growth deficiently was noted previously in *aro* mutants impaired in menaquinone biosynthesis [18]. Both the colony size and growth rate phenotypes were restored via genetic complementation. These findings are also supported by the transcriptional findings from Toledo-Arana et al. (2009) [19], that despite its proximity to the previously-studied *aro* genes, *lmo1930* is the penultimate gene in a menaquinone biosynthesis operon which includes the preceding ORFs *folE*, *lmo1932,* and *menH*, and is terminated by *ndk* (Figure 1). The extent to which mutants of other genes in this operon, and potentially also *aro* mutants, may also be impaired in esculin hydrolysis, LiCl tolerance and growth on selective media remains to be determined. While our findings exhibited some similarities to previous work, determining the role of *lmo1930* in menaquinone biosynthesis was not a major focus of the current study, and additional work would be required to conclusively establish that link.

In conclusion, the current study implicates *lmo1930*, a gene in an operon implicated in menaquinone biosynthesis, in two key phenotypes i.e., esculin hydrolysis and LiCl tolerance, required for the selective enrichment of *L. monocytogenes* in several most commonly-employed selective enrichment protocols. Our findings indicate that *L. monocytogenes* strains with mutations leading to inactivation of *lmo1930*, and possibly mutants of other genes involved in menaquinone biosynthesis, may evade detection in many of the selective enrichment protocols for *L. monocytogenes* that are currently employed. The incidence of such mutants in foods, the environment or illness remains currently unknown since they would not be detected as putative listeria with many enrichment protocols. Selective enrichment schemes that do not include LiCl and also utilize screening via molecular methods may be needed to ensure adequate surveillance of *L. monocytogenes* and other *Listeria* spp. in foods and the environment.
